# 1145. Real-world Effectiveness of Sotrovimab for the Early Treatment of COVID-19 in the US

**DOI:** 10.1093/ofid/ofac492.983

**Published:** 2022-12-15

**Authors:** Mindy M Cheng, Daniel C Gibbons, Helen Birch, Vishal Patel, Christopher F Bell, Myriam Drysdale, Sacha Satram, Carolina M Reyes

**Affiliations:** Vir Biotechnology, San Francisco, California; GSK, Brentford, Middlesex, England, United Kingdom; GSK, Brentford, Middlesex, England, United Kingdom; GSK, Brentford, Middlesex, England, United Kingdom; GlaxoSmithKline, Research Triangle Park, North Carolina; GSK, Brentford, Middlesex, England, United Kingdom; Vir Biotechnology, San Francisco, California; Vir Biotechnology, San Francisco, California

## Abstract

**Background:**

Sotrovimab, a monoclonal antibody (mAb), received Emergency Use Authorization (EUA) for the treatment of high-risk outpatients with symptomatic COVID-19. The study objective was to evaluate real-world effectiveness of sotrovimab (500 mg intravenous) in reducing the risk of mortality or hospitalization during the SARS-CoV-2 Delta and initial Omicron variant waves in the US.

**Methods:**

A retrospective analysis was conducted of de-identified patients (pts) diagnosed with COVID-19 (ICD-10: U07.1) from 9/1/2021 to 2/28/2022 in the FAIR Health FH NPIC^®^ claims database. Pts were divided into 2 cohorts based on HCPCS codes: treated with sotrovimab and not treated with any mAb (no mAb). Pts meeting EUA high-risk criteria were identified via pre-specified ICD-10-CM diagnoses in records ≤ 24 months prior to their first COVID-19 diagnosis (index date). Facility-reported mortality (referred to as “mortality”), all cause hospitalizations and intensive care unit (ICU) admissions within 30 days of index were identified. Chi-square test, ANOVA, or t-tests were performed to statistically compare cohorts at a 0.05 level of significance (2-sided). *P*-values were not adjusted for multiplicity. Multivariable logistic regression was conducted to estimate the risk of mortality or hospitalization within 30 days, adjusting for demographic and clinical factors.

**Results:**

Of the high-risk COVID-19 pts identified, 13,140 were treated with sotrovimab and 1,283,284 received no mAb therapy. Compared to the no mAb cohort, the sotrovimab cohort was older, had more baseline conditions, and were more likely to be female (all *p* < 0.0001). In the no mAb cohort, 0.59% died and 5.74% were hospitalized (of whom 30% in ICU). In the sotrovimab cohort, 0.08% died and 2.50% were hospitalized (of whom 15% in ICU). After adjusting for potential confounders, treatment with sotrovimab was associated with 83% reduced odds of 30-day mortality (OR: 0.17, 95% CI: 0.09-0.31) and 61% reduced odds of 30-day hospitalization or mortality (OR: 0.39, 95% CI: 0.35-0.43) among high-risk COVID-19 pts.

**Conclusion:**

In this US real-world observational study of high-risk COVID-19 pts during the Delta and initial Omicron waves, treatment with sotrovimab was associated with reduced odds of mortality and hospitalization compared to no mAb treatment.

**Disclosures:**

**Mindy M. Cheng, PhD**, Vir Biotechnology: Employee, Shareholder **Daniel C. Gibbons, PhD**, GlaxoSmithKline: Employee, GlaxoSmithKline GSK shareholder **Helen Birch, PhD**, GlaxoSmithKline: Employee, GlaxoSmithKline GSK shareholder **Vishal Patel, MSc**, GlaxoSmithKline: Employee, GlaxoSmithKline **Christopher F. Bell, n/a**, GlaxoSmithKline: Employee, GlaxoSmithKline GSK shareholder **Myriam Drysdale, PhD**, GlaxoSmithKline: Employee, GlaxoSmithKline GSK shareholder **Sacha Satram, PhD**, Vir Biotechnology: Employee, Vir Biotechnology Vir shareholder **Carolina M. Reyes, PhD**, Vir Biotechnology: Employee, Vir Biotechnology Vir shareholder.

